# A shared gene but distinct dynamics regulate mimicry polymorphisms in closely related butterfly species

**DOI:** 10.1098/rspb.2025.0966

**Published:** 2025-11-26

**Authors:** Sofia I. Sheikh, Meredith M. Doellman, Nicholas W. VanKuren, Phoebe Hall, Marcus R. Kronforst

**Affiliations:** ^1^ Department of Ecology & Evolution, The University of Chicago, Chicago 60637, IL, USA

**Keywords:** sex-limited polymorphism, evo–devo, gene expression, gene co-option, supergene

## Abstract

Sex-limited polymorphisms, like mating strategies in male birds and mimicry in female butterflies, are widespread across the tree of life and frequently adaptive. Considerable work has been done exploring why genetic variation resulting in sex-limited morphs is generated and maintained, yet little is known about their molecular and developmental genetic basis. In the butterfly genus *Papilio* (subgenus *Menelaides*), multiple species have female-limited polymorphism: females develop either mimetic or non-mimetic wing colour patterns, and each polymorphism is controlled by allelic variation at *doublesex* (*dsx*). Across several species, we found that alternative female morphs develop male-like colour patterns when we knock down *dsx* expression, establishing that *dsx* controls both sexual dimorphism and polymorphism. We also found that mimetic *dsx* alleles have unique spatiotemporal expression patterns between two species, *Papilio lowii* and *Papilio alphenor*. To uncover the downstream genes involved in the polymorphism between species, we compared RNA-seq data from *P. lowii* with previous work in *P. alphenor*. While some canonical wing patterning genes are differentially expressed in females of both species, the temporal patterns of differential expression are notably different. Our results indicate that, despite the putative ancestral co-option and shared use of *dsx* among closely related species, the mimicry switch functions through distinct underlying mechanisms.

## 1. Introduction

Despite the fact that sexes share the vast majority of their genomes, males and females can develop not only distinct phenotypes, but multiple alternative phenotypes suited to particular life histories and selective pressures [1,2]. Sex-limited polymorphism—the ability of one sex to develop multiple discrete morphs—thus represents a striking case of adaptive phenotypic variation. This type of adaptation appears in disparate taxa and phenotypes [2], including the alternative mating strategies of male beetles and fish, where some males adopt sneaker or fighter morphs [3,4], and female colour polymorphism in damselflies [5].

Studies characterizing the genetic control of sex-limited polymorphism have revealed that sex-specific trait variation can be achieved by encoding the polymorphism in the sex-linked genome. For example, in brood-parasitic cuckoos, females exhibit distinct plumage morphs based on their W chromosome haplotype, while the homogametic males develop a single monochromatic pattern [6]. In contrast, mapping studies have also identified examples in which the genetic basis of sex-limited polymorphism is both autosomal and Mendelian, with allelic variation at a single locus acting as a switch between alternative phenotypes. In some cases, such switch loci function as supergenes, wherein multiple linked genetic elements contribute to the coordinated regulation of a complex phenotype in a simple Mendelian manner [2,7]. This flurry of discoveries suggests that allelic variation at switch loci is sufficient to shape distinct developmental programmes that produce multiple complex phenotypes.

The molecular mechanisms through which alternative alleles actually switch developmental programmes to produce sex-limited polymorphism have been explored in only a handful of examples. In the brown anole, Feiner *et al.* suggest that alternative female colour pattern morphs develop as a result of changes in cell migration behaviour that may arise from alternative protein-coding sequences of *CCDC170* linked with oestrogen receptor *ESR1* [8]. In swordtail fish, the polymorphic false gravid spot in males mimics the pregnancy spot of female swordfish and is associated with alternative alleles of *kitlga* that have unique tissue- and allele-specific expression [9]. In ruffs, three male mating morphs are distinguished by hormonal differences associated with distinct alleles at *HSD17B2* [10]. Collectively, these examples illustrate that sex-limited polymorphism can develop by several potential mechanisms, including modifying gene expression patterns, altering protein function or regulating hormone levels.

**Figure 1.**
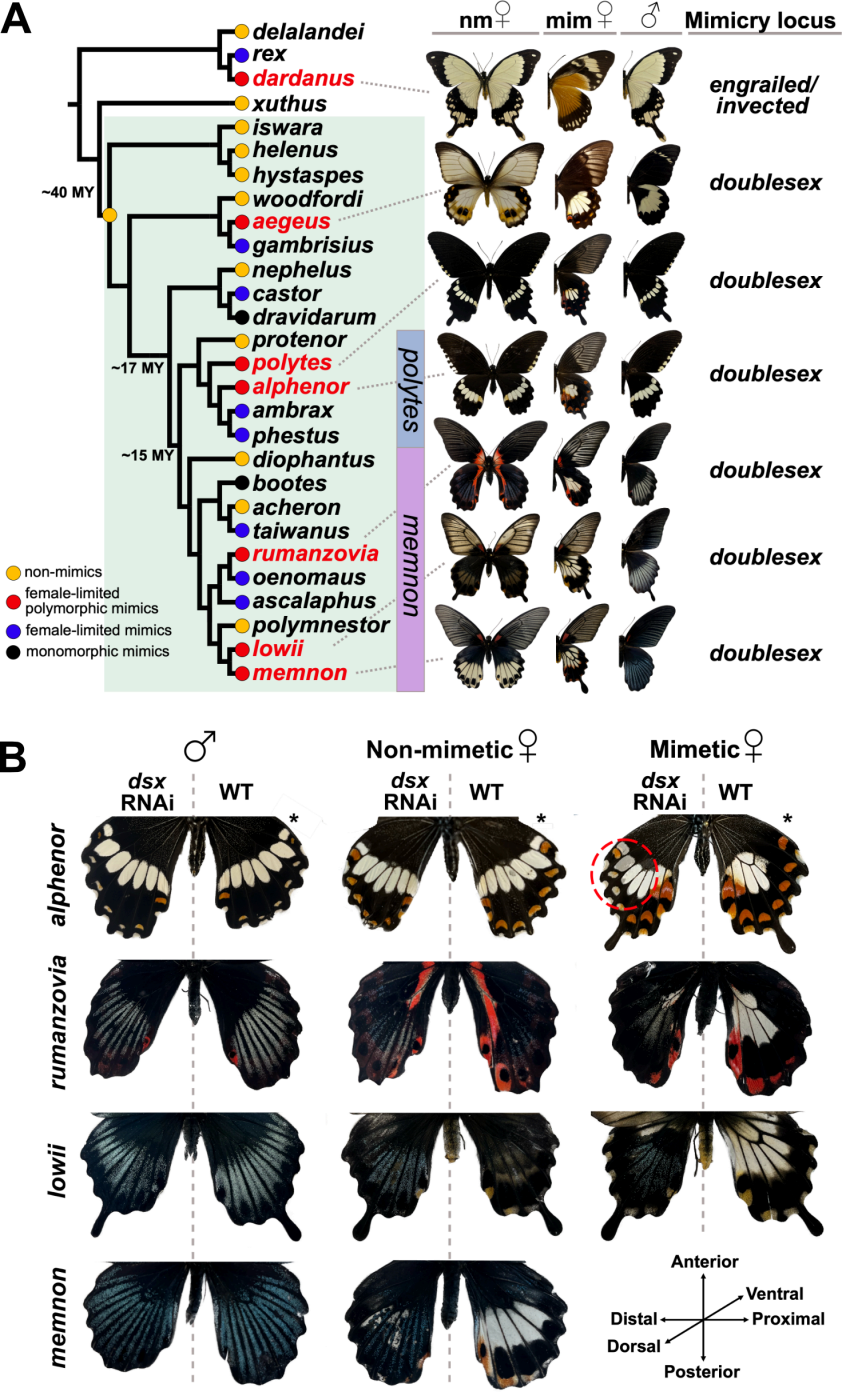
*dsx*-controlled mimicry polymorphism in *Papilio* butterflies. (A) Cladogram of a subset of *Papilio* species; *Menelaides* subgenus highlighted in green (24 out of 56 known species and two species groups shown), with species relationships, divergence estimates and mimicry state based on data from [11–13]. ‘nm’ refers to the non-mimetic female adult phenotype (homozygous for the recessive non-mimetic *dsx* allele), while ‘mim’ refers to the mimetic phenotype. Not all alternative colour patterns are shown for each species. See Methods for details about taxonomy. (B) *dsx* RNA interference (RNAi) phenotypes on the dorsal surface of genotypically mimetic and non-mimetic female and male hindwings. Untreated wild-type (WT) wings serve as a control to compare with the knockdown phenotype within an individual. *Papilio alphenor* RNAi individuals are from [14]. * Denotes individuals injected on the ventral surface, with the ventral surface of the butterfly shown here. The orientation key applies to all images without an asterisk and is shown here for the treated wing relative to the midline. For additional experimental and control RNAi individuals, see electronic supplementary material, figure S1; see [15] for *dsx* RNAi phenotype in mimetic *Papilio memnon*.

Beyond the limited number of functional case studies, explicit cross-species comparisons of the molecular and developmental control of sex-limited phenotype switching are missing. Yet, these comparisons are necessary to identify general principles on how developmental programmes and gene regulatory networks (GRNs) evolve to produce alternative phenotypes. *Papilio* swallowtail butterflies offer a powerful natural system to investigate the evolutionary developmental genetics of sex-limited polymorphism in a comparative context. Several species have evolved female-limited mimicry polymorphism (FLMP), wherein males develop a single non-mimetic wing colour pattern while females develop one of multiple discrete patterns, many of which mimic patterns of distantly related toxic species. In each case, the switch between female patterns is controlled by allelic variation at an autosomal locus (figure 1A). This female-limited polymorphism in *Papilio* butterflies is a classic example of supergene mimicry [11,16–20].

In some species, such as *Papilio dardanus*, *Papilio polytes* and *Papilio alphenor*, females can develop a male-like non-mimetic form or one of several discrete mimetic patterns. By contrast, in *Papilio memnon* and relatives, no female morph resembles the male colour pattern. In *P. dardanus*, an inversion in the regulatory region of the transcription factor *engrailed* is associated with a diversity of mimetic female morphs [21,22]. In the *Papilio* subgenus *Menelaides*, FLMP evolved through co-option of the conserved transcription factor *doublesex* (*dsx*) [11,19,20,23], which regulates sexual differentiation across insects [24,25]. In addition to performing its ancestral function, alternative *dsx* alleles determine the adult female colour pattern in at least six species (figure 1A).

The evolutionary origins of mimetic *dsx* alleles across the *Menelaides* subgenus remain unresolved, with equivocal evidence for a single ancestral origin or multiple independent origins. Across the entire subgenus, species with FLMP have extremely divergent mimetic and non-mimetic *dsx* haplotypes. Mimetic *dsx* alleles across species have unique amino acid substitutions, distinct signatures of linkage disequilibrium and varying structural features at the *dsx* locus [11,23,26]. Within the *polytes* group, species share a mimetic *dsx* allele, in addition to lineage-specific *dsx* haplotypes that produce additional mimetic female morphs [27]. The mimetic (dominant) alleles share an inversion containing the *dsx* region and have both coding and non-coding differences from the non-mimetic alleles. In the *memnon* clade (*Papilio rumanzovia*, *P. memnon and P. lowii*), no mimetic *dsx* alleles contain an inversion, and there are few to no coding sequence substitutions between the mimetic and non-mimetic haplotypes within a species [20,28]. These divergent patterns of sequence evolution suggest independent co-option of *dsx* in different lineages [26]. Alternatively, Palmer & Kronforst [11] suggested that the differences across mimetic *dsx* alleles may be the result of allelic turnover, wherein ancestral alleles are continuously replaced with derived variants, thus obscuring evidence of shared ancestry [11]. Whether *dsx* co-option evolved independently or ancestrally, the presence of a common switch gene across multiple species offers a critical look into how the same developmental machinery underlying sex-limited polymorphism has evolved since they last shared a common ancestor.

The functional basis of *dsx*-mediated mimicry has been extensively characterized in *P. polytes* and *P. alphenor*, with recent work highlighting the evolution of several novel *cis*-regulatory elements (CREs) that autoregulate mimetic *dsx* alleles leading to a spike of *dsx* expression in mimetic females during early pupal development [14,29]. This increased expression causes a suite of transcriptomic differences between mimetic and non-mimetic females throughout pupal wing development, ultimately resulting in the development of distinct adult patterns [14,30,31]. In *P. memnon*, *dsx* has been shown to control both mimetic and non-mimetic female colour patterns, whereas the mimetic *dsx* allele also causes mimetic abdominal pigmentation [15]. Additionally, mimetic *dsx* does not appear to show a dramatic spike of expression during early pupal development like it does in *P. alphenor* mimetic females [15].

The combination of functional studies in *P. polytes*, *P. alphenor* and *P. memnon* shows that *dsx* is required for the mimicry switch and offers a general understanding of *dsx* expression in the context of polymorphic wing colour patterning. What remains unknown is whether the *dsx* switch functions through the same developmental programmes to specify colour patterns in a sex-limited fashion. Characterizing this requires a comparative analysis of gene expression in colour pattern development across multiple species. We first tested the role of *dsx* expression in the adult colour patterns across species using RNA interference (RNAi), then characterized spatial and temporal patterns of *dsx* expression and its downstream effects in *P. lowii* with immunohistochemistry and RNA-seq. By directly comparing *dsx* expression and signatures of genome-wide differential expression between *P. lowii* and *P. alphenor* (which last shared a common ancestor *ca* 15 million years ago), we identify shared and unique elements of the wing developmental programmes underlying the *dsx* mimicry switch and provide insight into the molecular mechanisms by which sex-limited polymorphisms evolve.

## 2. Results

### (a) *dsx* has a conserved ancestral role specifying sexually dimorphic wing patterns

Genomic evidence suggests that *dsx* controls the colour pattern switch in all polymorphic *Menelaides* [11,19,20,23]. However, its role in wing colour patterning has been examined in only three species. In *P. alphenor* and *P. polytes,* knock down of *dsx* expression in mimetic females results in a mosaic wing pattern resembling the non-mimetic female or male colour pattern, whereas in *P. memnon*, mimetic females convert to the male form [14,19]. To confirm these results and test *dsx*’s role in wing colour patterning in additional polymorphic species, we used an RNAi electroporation approach to knock down *dsx* expression in the hindwing of three species in the *memnon* group: *P. rumanzovia, P. lowii* and *P. memnon*. In contrast to the *polytes* group, polymorphic species in the *memnon* group display strong sexual dimorphism between both female morphs and males (figure 1A). In these three species, RNAi caused females to develop a male-like colour pattern (figure 1B): treated regions of mimetic and non-mimetic females recovered a mosaic of the male-like adult colour pattern, exhibiting both blue structurally coloured scales and male-like patterning of those scales across the wing. These results align with recent findings from *dsx* RNAi in *P. memnon* [15]. In *P. alphenor*, *dsx* may also be required for sexually dimorphic patterning, but knock down in non-mimetic females causes only subtle effects on the adult colour pattern since they closely resemble males [14]. Males of species in the *memnon* group with mimetic or non-mimetic genotypes had slightly fewer blue scales in the *dsx* small interfering RNAs (siRNAs)-injected region compared with their wild-type (WT) wing and control males (electronic supplementary material, figure S1), though patterning was otherwise unchanged. Altogether, these results suggest that *dsx* performs a conserved function in controlling sexual dimorphism, while the different *dsx* alleles control the development of distinct female morphs.

### (b) DSX expression prefigures mimetic colour patterns but varies between species

**Figure 2.**
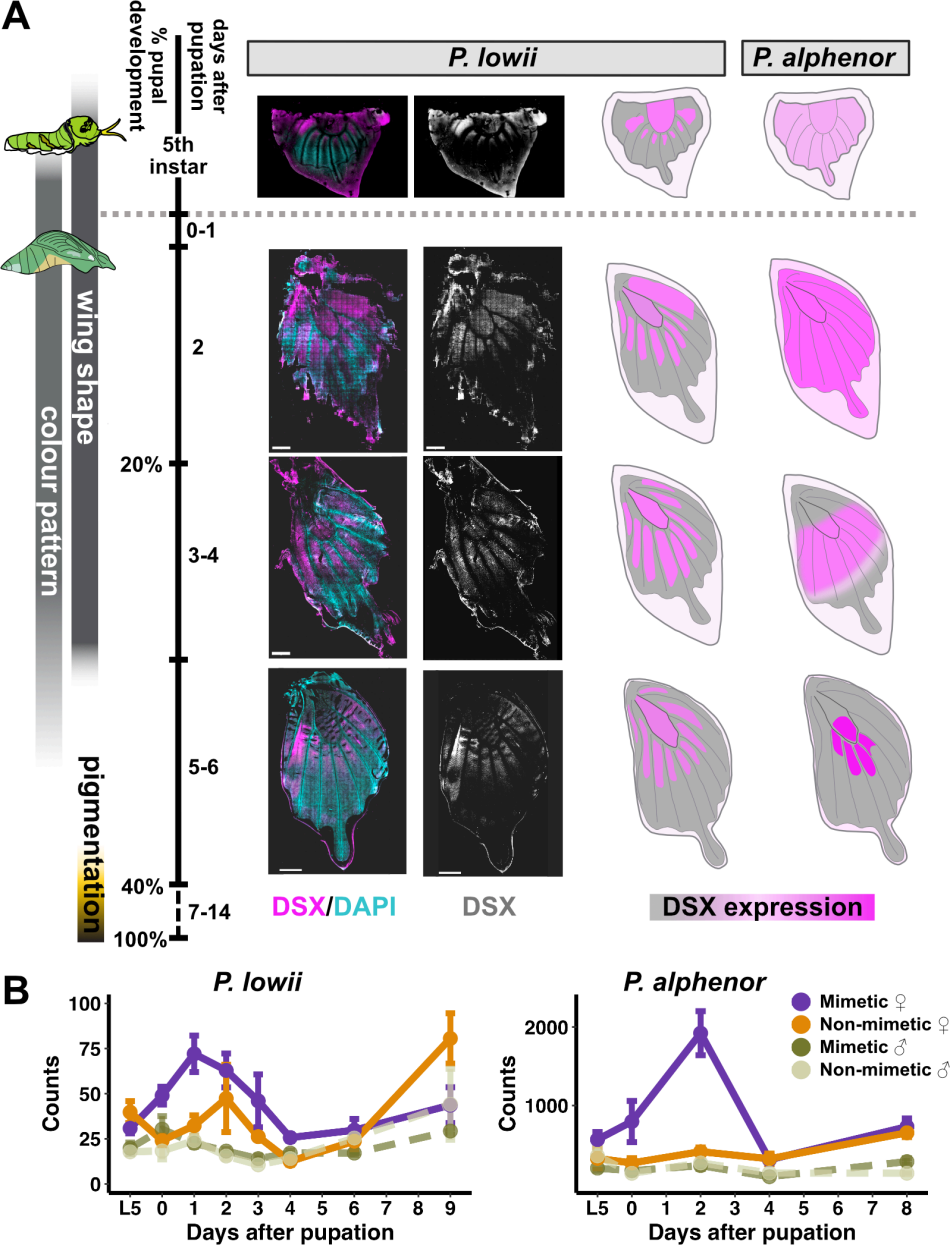
DOUBLESEX expression patterns in the hindwing of *Papilio lowii* and *Papilio alphenor*. (A) Left: anti-DSX staining from fifth-instar larval through mid-pupal development in hindwings from *P. lowii* mimetic female. Right: cartoon schematic of DSX expression in *P. lowii* antibody stains and known patterns in *P. alphenor* at the same developmental time points (*P. alphenor* schematic modified from [14]). Colour gradient reflects relative DSX expression (low to high). For additional staining images for *P. lowii* and late larval *P. alphenor*, see electronic supplementary material, figures S2 and S3, and [14]. Colour pattern includes cell fate specification and differentiation, while pigmentation involves biosynthesis and transport of pigments. .(B) *dsx* normalized quantifications across development in four groups of *P. lowii* (left) and *P. alphenor* (right).

After testing the role of *dsx* in female wing patterning, our second approach to assessing parallelism in the *dsx* mimicry switch was characterizing the expression patterns of DSX protein in the developing *P. lowii* hindwing. Previous work in *P. alphenor* showed that mimetic DSX has a dynamic expression pattern from early to mid-pupal development: initially, it is highly and uniformly expressed across the wing, and later it resolves to prefigure the central white patch in mimetic females [14]. Antibody staining in *P. lowii* revealed similar *dsx* enrichment in mimetic hindwings; however, its expression is spatially restricted earlier on during the fifth instar larval stage and maintained through early- to mid-pupal development (figure 2A). This expression pattern prefigures the medial light bands extending proximally and anteriorly in the mimetic female hindwing, with DSX localized in scale precursor and socket cells from early in pupal development. In mimetic females of *P. alphenor*, DSX is present in scale precursor, socket and epidermal cells across the hindwing during early pupal development and then gradually becomes spatially restricted to the scale and socket cells of the white patch in adult mimetic females (figure 2A). DSX also shows spatially restricted expression in non-mimetic *P. lowii* females (electronic supplementary material, figure S2), unlike *P. alphenor* non-mimetic females [14].

In addition to spatial expression patterns, we quantified *dsx* expression in *P. lowii* using bulk RNA-seq of hindwings from late larval through late pupal development (L5–P9), spanning pattern specification and pigmentation. We generated RNA-seq for *P. lowii* individuals homozygous for one *dsx* allele but included some heterozygous individuals for additional comparison (electronic supplementary material, table S2 and figures S4–S6). We compared these results with an existing and similar dataset in *P. alphenor* (NCBI SRA BioProject PRJNA882073 from [14]). Owing to highly divergent *dsx* alleles and genome-wide heterozygosity, we assembled a long-read Nanopore genome sequence for *P. lowii* (electronic supplementary material, table S3) and quantified gene expression for each species using its respective reference genome and annotation to avoid biasing quantification results. In *P. alphenor*, *dsx* is lowly expressed in all groups except mimetic females, where it undergoes a drastic and statistically significant increase of expression peaking at 2 days post-pupation. In contrast, *dsx* is lowly expressed across all *P. lowii* groups (figure 2B; electronic supplementary material, figure S7). In mimetic females, *dsx* showed a pulse of expression during early pupal development similar to *P. alphenor*; however, the difference in expression between mimetic and non-mimetic females was not statistically significant at any stage (overall false discovery rate (FDR) <0.01).

### (c) Unique transcriptomic signatures of the *dsx* mimicry switch

**Figure 3.**
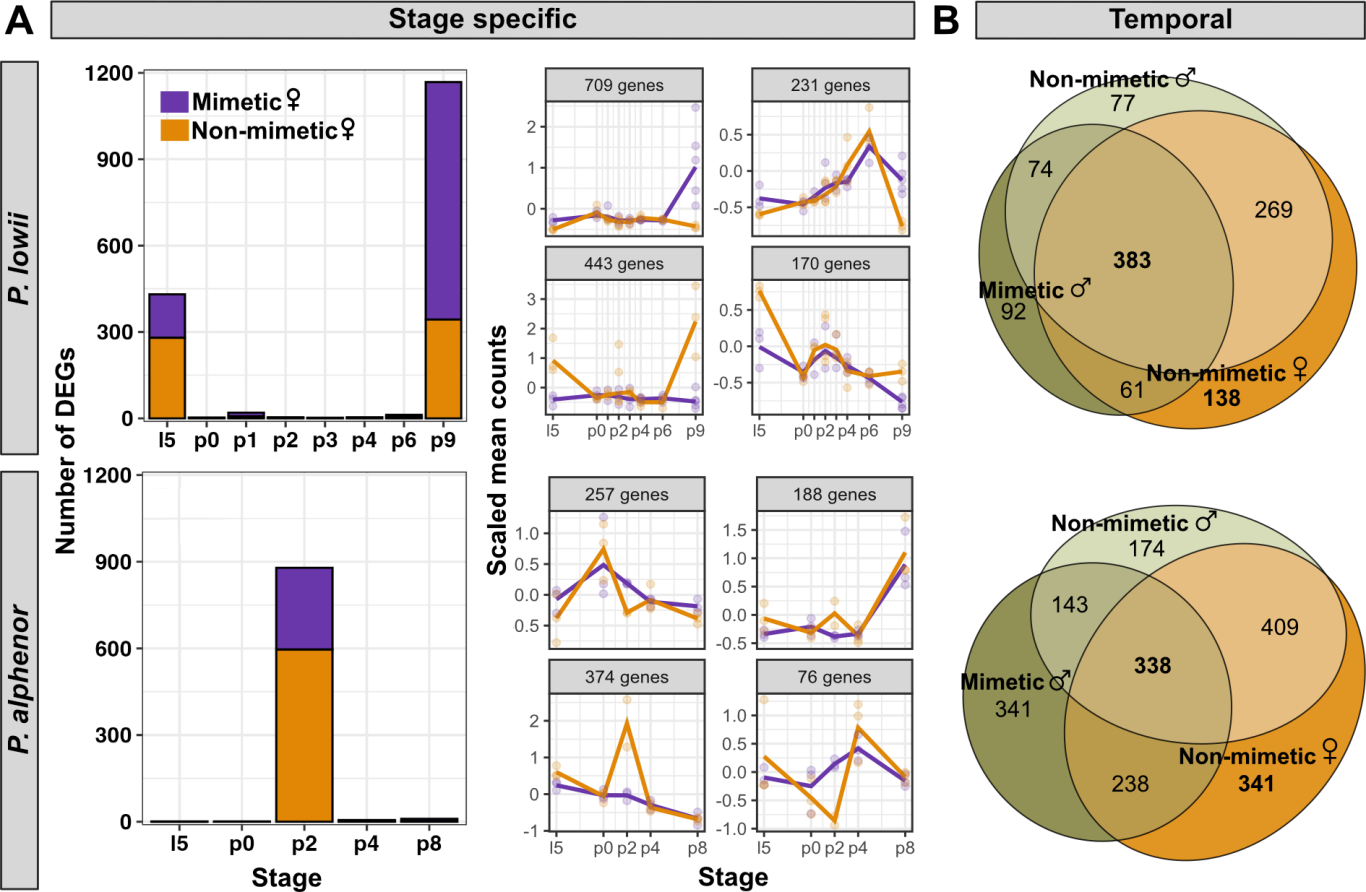
Differential gene expression underlying the mimicry switch in *Papilio lowii* and *Papilio alphenor*. (A) Left: genes differentially expressed and upregulated in either mimetic or non-mimetic females at each developmental stage. Stage refers to the number of days after pupation and is directly comparable between both species because they share total pupal development time. Right: mean expression profiles of four largest clusters of genes in each species identified using DESeq2. DEGs, differentially expressed genes. (B) Genes with significantly different temporal expression profiles in mimetic females relative to the other sex–genotype groups. The intersection represents genes unique to mimetic females, while the mimetic versus non-mimetic female comparison represents genes that are unique to the non-mimetic females.

Given the differences in mimetic DSX expression between *P. lowii* and *P. alphenor*, we next sought to characterize how the different *dsx* alleles shape downstream gene expression and regulatory networks underlying the mimicry switch in each species. First, we used two complementary approaches to identify differentially expressed genes (DEGs) in the RNA-seq datasets generated here for *P. lowii* and previously for *P. alphenor* [14]*.* We identified DEGs between mimetic and non-mimetic females at each stage using DESeq2. We found 1553 and 895 stage-specific DEGs in *P. lowii* and *P. alphenor*, respectively. In *P. lowii*, expression divergence was concentrated during late larval (L5) and late pupal development (9 days post-pupation; P9), whereas in *P. alphenor* almost all DEGs were found during early pupal development, coinciding with the spike of *dsx* expression at P2 (figure 3A, right). Moreover, the majority of stage-specific DEGs in *P. lowii* were upregulated in mimetic females with divergent expression trends during late larval and late pupal development, whereas in *P. alphenor* the opposite trend was observed and both female morphs had generally similar expression profiles at those same stages (figure 3A, left). While DESeq2 tested the effect of sex and *dsx* genotype on gene expression at each stage, we also tested for genes with distinct expression trends throughout the developmental time series using maSigPro. In total, 521 and 679 genes exhibited unique longitudinal expression patterns (overall FDR <0.01) in mimetic versus non-mimetic females of *P. lowii* (figure 3B, top) and *P. alphenor* (figure 3B, bottom). Some of these overlapped with DEGs identified using DESeq2; summing all unique genes from both approaches, we found a total of 1922 and 1491 DEGs in *P. lowii* and *P. alphenor*, respectively (electronic supplementary material, tables S4 and S5).

### (d) Parallelism at the effector gene level

**Figure 4.**
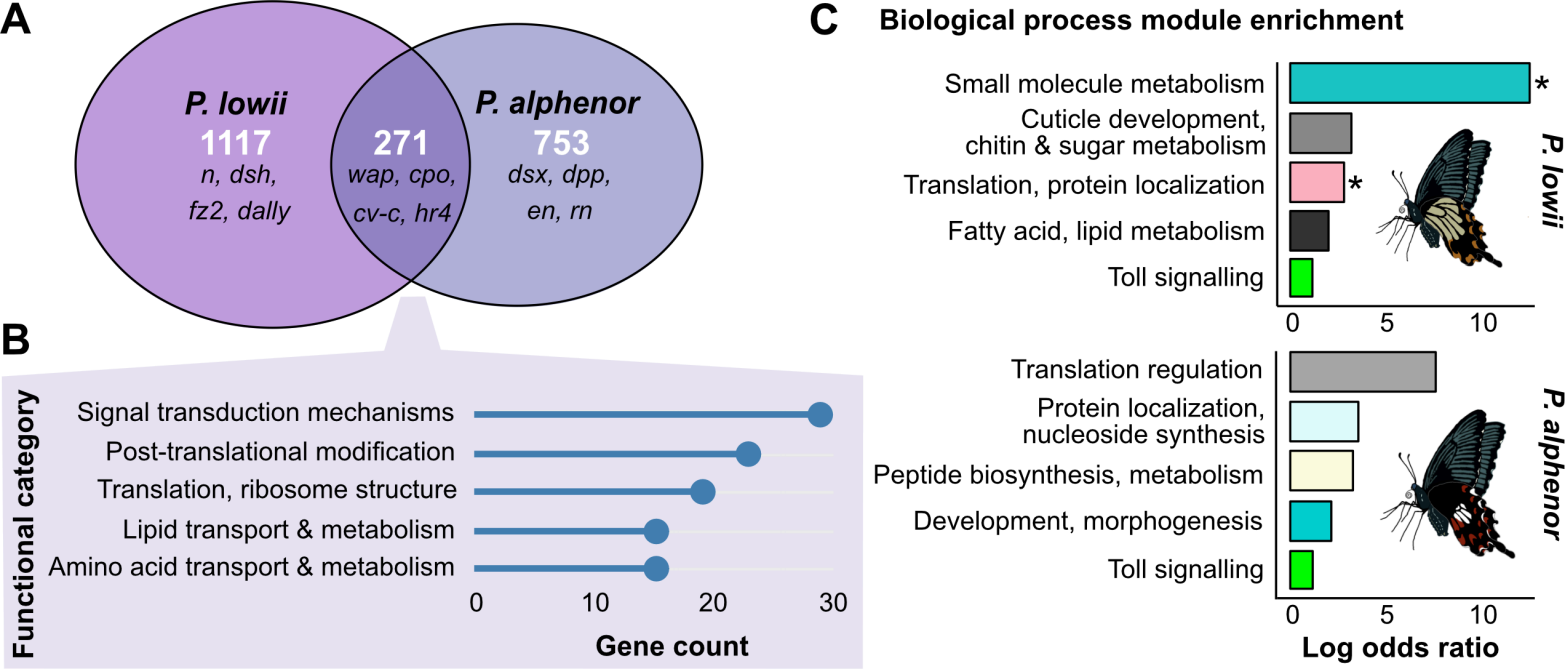
Developmental programmes and pathways associated with the mimicry switch. (A) Euler diagram of overlapping genes that are differentially expressed between mimetic and non-mimetic females of each species. Example of candidate genes based on literature listed for each set. Full results can be found in electronic supplementary material, table S6. (B) Top five Clusters of Orthologous Genes (COG) functional categories assigned to the overlapping differentially expressed genes (DEGs) in (A). (C) Gene ontology term enrichment for the top four co-expressed gene modules containing the highest number of DEGs in each species, and the single most strongly correlated module between both species. Colours reflect the module colour assigned by weighted gene co-expression network analysis (electronic supplementary material, figures S9 and S10). * Denotes modules that were also significantly correlated with the mimetic female group.

While the signatures of differential expression across development vary and may reflect differences in *dsx* regulation, the same downstream effector genes may still underlie both mimicry switches. Given the putatively ancestral co-option of *dsx* and shared function as a switch locus rewiring the wing regulatory network, we expected some overlap in DEGs. In particular, we expected to find shared signalling molecules and transcription factors involved in pattern specification. These genes act earlier in development, are more likely to be directly regulated by *dsx*, and therefore should show altered expression between female morphs of both *P. alphenor* and *P. lowii*. By contrast, downstream genes in terminal processes like pigment synthesis and deposition are likely to differ between species. To test this, we looked for overlap in the lists of DEGs. We first filtered for genes that were assigned an orthologue and that had expression data in both species. In this reduced dataset, 72% (1388/1922) and 69% (936/1490) of DEGs were assigned orthologous gene IDs in *P. lowii* and *P. alphenor*, respectively. Using these data, we identified genes that were significantly differentially expressed (DE) in both species, and ones that were uniquely DE within one species but still expressed in the other. We found that 19.5% of *P. lowii* DEGs were also differentially expressed in *P. alphenor* (figure 4A; electronic supplementary material, table S6). We performed a Fisher’s exact test (FET) based on genes that were differentially expressed in both species, unique to either species, or present in both transcriptomes but not differentially expressed and found a statistically significant association between DEGs in both species (*p* = 1.2e−11).

We next examined whether known regulatory and colour patterning genes were unique or shared between the two mimicry switches. Among genes that were uniquely DE in each species, several are key transcription factors and morphogens from conserved signalling pathways that have been repeatedly co-opted in wing patterning [32–35]. For example, three of the *P. lowii*-specific DEGs, *Notch, frizzled2* and *dishevelled* (a receptor for Notch signalling, and receptor and transducer for Wnt signalling, respectively), have ancestral functions in cell–cell communication for cell fate specification and segment polarity [36,37]. *Notch* has been co-opted to specify vein patterning and midline colour patterns [34], while *frizzled2* receives *WntA*, which can act to prefigure and specify boundaries of adult colour patterns [38]. Interestingly, while these Wnt signalling components were not DE in *P. alphenor*, other key players were, including the regulator *shaggy*. Additionally, the *dsx* mimicry switch in *P. alphenor* appears to be mediated by several transcriptional co-regulators and targets like *yorkie* and *decapentaplegic* [14]. Interestingly, we found that though *engrailed* was DE in only *P. alphenor*, immunohistochemistry results suggest that it has a similar function in *P. lowii* (electronic supplementary material, figure S8).

Several of the shared genes also play key roles in early wing development. For example, the RNA-binding protein gene *couch potato* is enriched in precursor and developing scale and socket cells in wings of the butterfly *Bicyclus anynana* [39]. Similarly, c*rossveinless-c* encodes a BMP-binding protein required for wing vein morphogenesis [40], and *wings apart* inhibits the transcriptional repressor *capicua* in order to promote tissue growth and patterning, including of insect wings [41]. Overall, among the 271 DEGs that were shared in the mimicry switch between both species, Clusters of Orthologous Genes (COG)-based functional annotation highlighted enrichment for translational processes and signal transduction mechanisms (figure 4B), suggesting that mimetic *dsx* likely has a drastic effect on cell specification and not the downstream/terminal processes in wing phenotype development.

### (e) Parallelism at the gene regulatory network level

Finally, we compared the GRNs involved in the *dsx* mimicry switches using weighted gene co-expression network analysis (WGCNA). Each module was assigned a colour at random and represents clusters of genes with strongly correlated expression profiles, thus potentially corresponding to biologically functional units []. We found 23 modules in *P. lowii* and 31 in *P. alphenor*. After identifying modules, we tested for correlations between each module with various traits (stage, sex, genotype and group), as well as modules that were significantly enriched with DEGs. In *P. lowii*, 14 modules were significantly enriched or deficient in DEGs (FET, Benjamini–Hochberg (BH)-corrected *p*‐value <0.05). Of these, five modules were significantly correlated with mimetic females; that is, the module’s gene expression patterns were significantly associated with the group (BH-corrected FET *p*‐value <0.01; electronic supplementary material, figure S9 and table S7). In *P. alphenor*, 17 modules showed significant enrichment or deficiency of DEGs, of which two modules were significantly negatively correlated with mimetic females (electronic supplementary material, figure S10 and table S7). We first examined the most common processes from the top Gene Ontology (GO) terms of the first four modules that were most significantly enriched with DEGs in each species. We found that modules most enriched for DEGs in each species were associated with some shared GO terms, including translation, protein localization, and metabolism (figure 4C).

We sought to compare whether network modules were preserved across species and to identify orthologous modules. Using the WGCNA ModulePreservation function, we found that overall network topologies (relationships, connectivity and expression patterns of genes) were highly preserved between species. This is expected because the networks and modules for each species were generated from expression data from hindwing tissue, and the biological processes involved in wing development are likely to be conserved. However, despite this preservation in network topologies, the module compositions were considerably different in terms of gene membership between species, and we therefore were only able to identify orthologous modules for six out of 23 *P. lowii* modules using a combination of the preservation score and gene membership count (electronic supplementary material, figure S11). Of these, two were deficient in DEGs between and associated with cell division, hormone regulation and lipid metabolism GO terms. A third orthologous module had GO terms associated with toll signalling (figure 4C, green). As part of its role in immune defence, toll signalling is involved in melanization, and this process is also used to control stripe patterning in *Bombyx mori* caterpillars [42]. Additionally, several toll signalling genes are upregulated in other butterfly wing patterns, including eyespots in *B. anynana* [43], wing spots in *Pieris candida* [44] and red spots in mimetic *P. polytes* [45].

## 3. Discussion

Here, we compared the role of *dsx* in orchestrating the development of discrete, complex female morphs among closely related species in the *Menelaides* subgenus. This system presents a unique case of parallelisms at multiple scales—the phenotypic state of mimicry; sexual dimorphism and female polymorphism; the switch gene dictating the adult female colour pattern; regulation of switch gene expression, and potentially its downstream consequences on wing patterning. To begin dissecting the different levels of parallelism, we first characterized the role of *dsx* in wing colour patterning of polymorphic species. Our RNAi results show that sexual dimorphism in wing patterning is governed by *dsx,* consistent with its role in controlling insect sexual dimorphism [24,25]. Importantly, in addition to this role, it is allelic variation at *dsx* that determines which colour patterns females develop. Across these species, *dsx* functions as a switch gene, which can coordinate the expression of shared or species-specific downstream genes to develop discrete morphs [46]. Thus, *dsx* functions by ‘toggling’ between alternate wing pattern developmental programmes.

To further characterize how the *dsx* switch gene functions across species, we focused additional functional experiments on *P. lowii* owing to its evolutionary distance from the well studied species *P. alphenor*. In comparing *dsx* expression from our experiments in *P. lowii* with previous work in *P. alphenor* [14], our results suggest that the mimetic alleles are expressed in notably different patterns, in terms of both their absolute levels and their spatial patterns across the developing hindwing. Moreover, the non-mimetic *dsx* alleles appear to vary in their expression between species. These results may suggest that *dsx* was convergently co-opted in mimicry polymorphism evolution two or more times. Alternatively, our findings likely indicate *cis*-regulatory divergence in the *dsx* supergene following co-option in the common ancestor, in concordance with the idea of allelic turnover from an ancestral mimetic *dsx* in *Papilio* [11]. Future work on the regulatory landscapes of different *dsx* alleles across *Menelaides* is needed to identify the shared and lineage-specific repertoire of CREs that instruct when and where DSX is expressed—both between morphs within a species and among mimetic female morphs across polymorphic species.

Beyond the switch gene itself, we examined transcriptomic changes due to mimetic *dsx* alleles in *P. alphenor* and *P. lowii*. We observed surprising differences in the developmental windows at which gene expression was significantly altered between mimetic and non-mimetic females, suggesting that unique developmental trajectories underlie the mimetic female phenotype across these species despite the shared use of *dsx*. Nonetheless, we also identified some critical cell fate specification and patterning genes that were differentially expressed in both species, suggesting that both species may share some proximate functional mechanisms of mimetic wing patterning by *dsx*. These genes may be directly regulated by *dsx* or represent critical nodes of wing developmental networks that facilitate phenotype switching among females. Interestingly, roughly a quarter of the genes that were differentially expressed in both species are also differentially bound by DSX in *P. alphenor* mimetic females compared with non-mimetic females [29] and represent promising genes to target for future functional characterization.

Overall, there appear to be considerable lineage-specific differences despite both species being closely related and using the same switch gene for FLMP. While constraints exist in wing GRNs [47], the GRNs themselves are dynamic throughout development [48]. The opposing windows of differential expression that we observed between *P. lowii* and *P. alphenor* suggest that DSX may be modifying expression of distinct elements of wing developmental GRNs. Additionally, wing shape, pattern and colour differ between species, and this could be responsible for some of the observed molecular differences. For example, in *P. memnon* and *P. alphenor*, tails are polymorphic and only develop in mimetic females, whereas the tail is a monomorphic structure in *P. lowii*. Similarly, abdominal pigmentation is only observed in mimetic females of *P. memnon*. These differences suggest the presence of modifier genes that vary between species. While supergene theory emphasizes the physical linkage of functionally relevant genetic elements [7,49], there may also be unlinked genes that epistatically interact with the mimetic allele to modify or enhance the mimicry phenotype [50]. These modifier loci, whether linked or unlinked to the switch locus, remain unknown beyond candidate genes in *P. alphenor* [29]. Among the unique DEGs we identified between species, some may act as species- and morph-specific modifier genes required to execute each mimicry switch.

Another potential explanation for the observed divergence between *P. lowii* and *P. alphenor* is developmental systems drift, wherein the genetic and regulatory bases of homologous traits diverge even among closely related species without changing the trait itself [51]. One of the most obvious cases of phenotypic invariance coupled with divergent developmental underpinnings is sex determination and differentiation across taxa [52]. For example, in dipteran flies, the sex determination hierarchy diverges in upstream signals (*Sxl* in *Drosophila* versus *F* in *Musca*) while the downstream effector (*dsx*) is conserved, maintaining the same developmental outcome despite lineage-specific genetic interactions [51,53]. Similarly, in the recent radiation of *Heliconius* butterflies, convergent wing patterns have evolved through distinct WntA-mediated developmental pathways [54]. In *dsx*-mediated mimicry, it is possible that an ancestral co-option of *dsx* was followed by lineage-specific drift in the developmental genetics underlying female polymorphism. This may be reflected by the considerable overlap yet differences in DEGs, and the observation that different genes in known wing specification and patterning pathways are differentially expressed within each species. Comparing the direct targets of mimetic DSX and genetic manipulations of DSX targets across species will help test the relative roles of species-specific modifiers and developmental systems drift in the developmental differences among sex-limited polymorphic species.

Finally, we note that bulk RNA-seq approaches to characterizing homology in molecular mechanisms and GRNs have limitations. The loss of cellular-level resolution can obscure critical signals and differences between cell types [55]. Additionally, the lack of conservation in gene expression patterns across species may not indicate functional divergence [55]. Cross-species comparisons may also suffer from technical challenges, including identifying orthologous genes and reference genome differences. Therefore, we may be missing some signatures of shared functional genes/pathways in our comparative RNA-seq analyses. However, we expect that, using our approach, we detected the most strongly differentially expressed genes in each species and that those genes play a role in defining and executing the alternate developmental programmes within each species. Thus, the comparison between species enables us to begin describing the shared and unique elements of the developmental genetics underlying sex-limited polymorphism. Future work leveraging single-cell approaches is poised to allow a finer-scale dissection of the molecular parallels between these polymorphic species.

## 4. Methods

### (a) Notes on taxonomy

The taxonomic status of some *Papilio* species has recently been revised. For this manuscript, we used the previous species designations of *P. rumanzovia, P. lowii* and *P. memnon*, but note the following taxonomic changes based on Condamine *et al.* [56]: the authors suggest that *P. rumanzovia* is a subspecies of *Papilio deiphobus* and *P. lowii* is a subspecies of *P. memnon*. Additionally, the mainland and island taxa of *P. memnon* were historically designated as a single species but now represent two distinct species based on their geography: *Papilio agenor* (mainland) and *P. memnon* (Southeast Asian islands). The *P. memnon* used in this study were from Thailand and therefore would be considered *P. agenor* under the new taxonomy*.*

### (b) Butterfly care and development staging

Butterfly pupae were provided by butterfly breeders in the Philippines (*P. lowii* and *P. rumanzovia*) and Thailand (*P. memnon*) and grown in the greenhouse at the University of Chicago. After emergence, adult butterflies were separated into male and female cages. We collected a single leg from each live adult and used it to genotype *dsx* alleles with custom TaqMan assay probes (Thermo Scientific). Genotype results were used to set up single or multi-pair homozygous crosses in mesh cages with *Citrus* shrubs. From the cages, we collected prepupae each morning and stored them in an incubator matching their natural environment: 70% relative humidity, 25°C, and 16 h light : 8 h dark cycle. p0 pupae were defined as being between 12 and 24 h after pupation.

### (c) RNA interference

We knocked down *dsx* expression using the RNAi protocol from [57] with modifications described in [14]. Prepupae were collected from the cross cages in the greenhouse, and pupation was closely monitored so that RNAi experiments could be carried out at the time of pupation when the cuticle was still labile. For injections, we designed Dicer substrate siRNAs (DsiRNAs) using IDT’s DsiRNA design tool and a *P. lowii* genome (see below), identifying off-target sites with primer-BLAST (electronic supplementary material, table S1). We injected 2 µl of 100 µM DsiRNA or 1× phosphate-buffered saline (PBS) as a control into the ventral surface of the left hindwing close to the discal cell and between vein landmarks CU1 and M3. To improve cell permeability for DsiRNA uptake, we covered the injection site with PBS and electroporated the site with the positive pole (red electrode) positioned on the dorsal surface. The hindwing cuticle cover was then folded back, and the injected pupae were placed into a Petri dish with moist paper towels. We stored the Petri dishes in the same incubator with settings described above, and pinned and imaged the adult butterflies.

### (d) Antibody staining

We characterized DSX spatial expression pattern in *P. lowii* using a polyclonal antibody raised against the closely related species *P. alphenor* [14]. The DSX antibody was raised against the protein encoded by exons 1 and 2 (217 residues) of *P. alphenor*, which constitute more than 80% of the full protein and are present in all isoforms. These exons are highly conserved in *P. lowii*, with a 94% identity between *P. lowii* and *P. alphenor* mimetic *dsx* alleles. For staining, hindwings across different developmental stages were dissected out in 1× room temperature PBS following the protocol described in [14]. We used the rabbit anti-Dsx antibody at a 1 : 250 dilution and co-stained it with either 4′,6-diamidino-2-phenylindole (DAPI) or Hoescht as a control. To image the dorsal surface of whole mounted wings, a 20× objective and a *Z*-stack/tile scan was used on a Zeiss LSM 710 confocal microscope at the University of Chicago. Images were then converted to maximum intensity projection and stitched in Zen (Carl Zeiss Microscopy, Jena, Germany). We initially processed and scaled images in Fiji [58] and then imported them to Inkscape for brightness and contrast adjustment.

### (e) RNA-seq sampling, extraction and sequencing

We collected three replicates per developmental stage, sex and *dsx* genotype combination for RNA-seq. Pupae were collected from the incubator at approximately the same time each day to reduce developmental variance between biological replicates. Each replicate consisted of two hindwings dissected from a single pupa and stored in RNAlater (Ambion) at −80°C until extraction. We extracted total RNA using TRIzol (Ambion). TruSeq library preparation using poly-A selection and sequencing (PE100) on a NovaSeq X was done by the Functional Genomics Facility at the University of Chicago (electronic supplementary material, table S2).

### (f) *Papilio lowii* genome sequencing, assembly and annotation

We extracted high molecular weight genomic DNA (HMW gDNA) from the thorax of a freshly killed *P. lowii* female homozygous for the mimetic *dsx* allele using the QIAgen Genomic-tip G-100 kit. Extractions followed the manufacturer’s instructions, except we incubated chopped fresh tissue in lysis buffer overnight in a thermomixer at 50°C and 200 r.p.m. before purification. We then constructed Oxford Nanopore sequencing libraries using the ONT Ligation Sequencing kit (LSK-110) and eliminated fragments <10 kb using the PacBio SRE XS kit before sequencing on a MinION Mk1b and R9.4.3 flow cells to 30–40× coverage.

We called bases using Guppy and super high-quality base calling (dna_r9.4.1_450bps_sup.cfg), then assembled the genome using these raw reads and Flye v.2.9.1 [59] with default settings with expected genome size set to 250 Mb. The initial Flye assemblies were each polished using the Guppy basecalls and Medaka v1.7.2 (medaka_consensus) with the appropriate error model (r941_min_sup_g507). We then purged duplicates using purge_dups v.1.2.5. We used a custom repeat library for *P. alphenor* [14], the RepBase 20181026 ‘Arthropoda’ database [60], and Dfam 20181026 database [61] to identify and mask repeats genome-wide in both assemblies using RepeatMasker [62]. The final assembly comprised 251.9 Mb in 751 scaffolds (N50 1.6 Mb). BUSCO v.5 using the OrthoDB v.10 Endopterygota database [63] showed the assembly contained 98.7% complete (0.5% duplicated), 0.1% fragmented and 1.2% missing single-copy orthologs (SCOs).

We annotated the *P. lowii* assembly using EvidenceModeler (EVM) v1.1.1 [64]. We first assembled a high-quality transcript database using PASA 2.4.1 [64] and the PE100 data generated here. After adapter trimming, we performed *de novo* assembly using Trinity v.2.10.0 [65] and genome-guided assembly using StringTie v.1.3.3 [66]. RNA-seq data were also mapped to the assembly using STAR 2.6.1d [67], and the resulting alignments were used to generate genome-guided assemblies with Trinity and StringTie v.1.3.3 [66]. We combined *de novo* and genome-guided assemblies using PASA 2.4.1 [68]. Evidence for protein-coding regions came from mapping the UniProt/Swiss-Prot (2020_06) database and all Papilionoidea proteins available in NCBI’s GenBank nr protein database (accessed June 2020) using exonerate [69]. We identified high-quality multi-exon protein-coding PASA transcripts using TransDecoder (transdecoder.github.io), then used these models to train and run Genemark-ET 4 [70] and GlimmerHMM 3.0.4 [71]. We also predicted gene models using Augustus 3.3.2 [72], the supplied *heliconius_melpomene1* parameter set, and hints derived from RNA-seq and protein mapping above. Augustus predictions with >90% of their length covered by hints were considered high-quality models. Transcript, protein and ab *initio* data were integrated using EVM with the weights in electronic supplementary material, table S3.

Raw EVM models were then updated twice using PASA to add untranslated regions (UTRs) and identify alternative transcripts. Gene models derived from transposable element proteins were identified using BLASTp and removed from the annotation set. We manually curated the *dsx* region. The final annotation comprised 21 733 genes encoding 30 844 protein-coding transcripts, containing 97.6% complete and missing 1.5% of Endopterygota single-copy orthologues according to BUSCO v.5 and OrthoDB v.10 [63]. We functionally annotated protein models using eggNOG’s emapper-2.0.1b utility and the v.2.0 eggNOG database [73].

### (g) Differential expression analysis

We used the annotated genome to quantify transcript expression levels in Salmon v1.10.0 with bias correction [74]. Quantification results were imported and normalized in R using tximport v.1.34 [75] and filtered for lowly expressed genes, which were defined as genes with an average normalized count below 5 across all samples. From filtered, normalized data, we used mapping rate, classical principal coordinates analysis (PCA) (95% CI) and robust PCA using the PcaGrid function in rrcov v.1.7-6 [76] to identify outlier samples. After removing outliers, we used filtered, normalized gene-level quantification data in DESeq2 v.1.46 [77] to identify DEGs at each stage in pairwise comparisons of the female groups (mimetic female versus non-mimetic females). We also used maSigPro v.1.78 [78] to identify DEGs, defined as genes with significantly different temporal expression profiles in mimetic females compared with all other genotype–sex groups (non-mimetic females, mimetic males, non-mimetic males). Significant genes were first selected using a *q*-value cutoff of 0.01 in the p.vector() function. Subsequently, variable selection was performed based on a *p*-value threshold of 0.05 in T.fit(), and genes with good model fits were defined as those with *R*² >0.8. To identify orthologous genes between the two species, we used OrthoFinder v.2.5.5 with the set of longest isoforms for each species and default settings [79]. From the resulting single-copy orthogroups, we retained only 1 : 1 orthologues (10231 genes—52% of *P. alphenor* genes, 47% of *P. lowii* genes) to detect overlapping DEGs. We also constructed a second set of ‘relaxed’ 1 : 1 reciprocal matches (849 additional genes); these orthologues were defined as gene pairs that were reciprocal best hits in the pairwise orthology tables, including cases from multi-copy orthogroups where the two species still had a unique reciprocal partner. Of the genes expressed in the wing transcriptome of *P. lowii* and *P. alphenor* (11701 and 11307 genes, respectively), approximately 70% had an orthologue assignment in both species.

### (h) Co-expression network reconstruction and module preservation

Using RNA-seq data from *P. lowii* generated in this study, along with previously published RNA-seq data from *P. alphenor* [14], we constructed hindwing developmental gene co-expression networks for both species. For each species separately, networks were built using WGCNA v.1.73 [80] with normalized gene-level expression data, and both adjacency and topological overlap matrices were generated using signed Pearson correlation coefficients. To examine the relationships between gene modules and traits (stage, sex, genotype, sex–genotype combination), Pearson correlations were calculated between each module eigengene and trait variable, with corresponding *p*-values determined using Student’s correlation test. We then tested each module for enrichment or depletion of DEGs using Fisher’s exact test; modules with adjusted *p*-values <0.01 were considered significantly enriched or depleted in DEGs. We then conducted GO enrichment analysis for each module using topGO v.2.58 [81]. GO term assignments were derived from eggNOG-mapper v.2.1.12 annotations [73]. We tested for enrichment of biological process terms using Fisher’s exact test, with a minimum node size of five genes and an adjusted *p*-value threshold of 0.01. The top 50 enriched terms were retained for each module. After completing network reconstruction for each species, we used the ModulePreservation function in WGCNA to identify orthologous modules between both species. For our input datasets, we used the full normalized expression data and the gene assignments to modules from each species.

## Data Availability

Illumina RNA sequencing data and the genome assembly and annotation are publicly available in the National Center for Biotechnology Information (NCBI) under BioProject PRJNA1230174. Anti-DSX antibody is available from the authors upon request. Supplementary material is available online [82].
